# Audiovisual Modulation in Music Perception for Musicians and Non-musicians

**DOI:** 10.3389/fpsyg.2020.01094

**Published:** 2020-05-29

**Authors:** Marzieh Sorati, Dawn Marie Behne

**Affiliations:** Department of Psychology, Norwegian University of Science and Technology, Trondheim, Norway

**Keywords:** musicians and non-musicians, musical experience, auditory, audiovisual, music perception, inter-trial phase coherence (ITPC), event-related potential (ERP)

## Abstract

In audiovisual music perception, visual information from a musical instrument being played is available prior to the onset of the corresponding musical sound and consequently allows a perceiver to form a prediction about the upcoming audio music. This prediction in audiovisual music perception, compared to auditory music perception, leads to lower N1 and P2 amplitudes and latencies. Although previous research suggests that audiovisual experience, such as previous musical experience may enhance this prediction, a remaining question is to what extent musical experience modifies N1 and P2 amplitudes and latencies. Furthermore, corresponding event-related phase modulations quantified as inter-trial phase coherence (ITPC) have not previously been reported for audiovisual music perception. In the current study, audio video recordings of a keyboard key being played were presented to musicians and non-musicians in audio only (AO), video only (VO), and audiovisual (AV) conditions. With predictive movements from playing the keyboard isolated from AV music perception (AV-VO), the current findings demonstrated that, compared to the AO condition, both groups had a similar decrease in N1 amplitude and latency, and P2 amplitude, along with correspondingly lower ITPC values in the delta, theta, and alpha frequency bands. However, while musicians showed lower ITPC values in the beta-band in AV-VO compared to the AO, non-musicians did not show this pattern. Findings indicate that AV perception may be broadly correlated with auditory perception, and differences between musicians and non-musicians further indicate musical experience to be a specific factor influencing AV perception. Predicting an upcoming sound in AV music perception may involve visual predictory processes, as well as beta-band oscillations, which may be influenced by years of musical training. This study highlights possible interconnectivity in AV perception as well as potential modulation with experience.

## 1. Introduction

Music is regularly heard without seeing the movements producing it, however, music perception is cross-modal and not solely based on auditory music perception. Body gestures, facial expressions, and especially finger and hand movements that require a high level of temporal and spatial accuracy are also involved in music perception. This information provides visual cues which assist the intelligibility of music (Thompson et al., 2005; Molnar-Szakacs and Overy, 2006; Repp and Knoblich, 2009; Behne and Wöllner, 2011; Platz and Kopiez, 2012; Maes et al., 2014), as has similarly been observed for speech (Klucharev et al., 2003; Schwartz et al., 2004; Van Wassenhove et al., 2005; Stekelenburg and Vroomen, 2007; Arnal et al., 2009; Pilling, 2009; Paris et al., 2013, 2016a,b; Baart and Samuel, 2015; Biau and Soto-Faraco, 2015; Hsu et al., 2016). For example, in audiovisual (AV) speech a talker's facial articulations begin before the sound onset, providing a perceiver with potential cues to predict the upcoming speech sound, and thereby enhance AV speech perception relative to the audio only (Besle et al., 2004; Schwartz et al., 2004; Paris et al., 2013). Likewise, in a piano performance, visual information from finger and hand movements can signal the timing of musical events (Thompson et al., 2005), and depending on the key being played, potentially also provide predictive information about the frequency of the upcoming sound (Maes et al., 2014).

Electrophysiological studies from AV perception have demonstrated that visual information from facial movements, beginning before onset of the auditory speech, can predict an upcoming speech sound and modulate AV speech perception (e.g., Stekelenburg and Vroomen, 2007; Paris et al., 2017). This modulation implies that measures of early event-related potentials (ERPs), such as N1 and P2, would be lower for AV speech compared to the condition with auditory perception (Baart, 2016). Although the N1-P2 waveform is an auditory evoked response commonly sensitive to variations in the physical features of auditory stimuli (Näätänen and Winkler, 1999; Tremblay et al., 2006), previous research (Huhn et al., 2009) has shown that, as a result of different spatially underlying mechanisms, N1, and P2 display different scalp distributions. N1 is the negative anteriorly-distributed component occurring around 100 ms in response to abrupt acoustical changes. One of the primary sources of N1 is the medial transverse temporal gyri (Tan et al., 2016). The N1 waveform is sensitive to attentional variations (Näätänen and Picton, 1987; Näätänen et al., 2011; Lange et al., 2013; Paris et al., 2013) and is influenced by the predictability of the upcoming sound based on corresponding visual cues, such as lip movements in speech (Paris et al., 2017), through a direct circuitry from visual to the auditory areas (Arnal et al., 2009). N1 is also modulated by inter-individual differences (Liem et al., 2012; Tan et al., 2016). The N1 waveform is followed by a positive fronto-centrally distributed P2 component which occurs 200 ms after the onset of auditory stimuli (Pratt, 2011) and is strongly associated with the auditory association areas in the brain (Bosnyak et al., 2004; Kühnis et al., 2014). In AV perception, through a feedback via superior temporal sulcus, visual information congruent with an auditory signal can lead to suppression of amplitude and latency of P2 (Van Wassenhove et al., 2005; Arnal et al., 2009; Paris et al., 2016b).

Amplitude and latency reduction for N1 and P2 in AV perception is not limited to AV speech perception (Stekelenburg and Vroomen, 2007, 2012; Baart et al., 2014). AV modulation has also been observed in studies with ecologically valid stimuli such as clapping hands and tapping a spoon against a cup (Stekelenburg and Vroomen, 2007; Vroomen and Stekelenburg, 2010) and artificial stimuli, such as moving bars (Paris et al., 2016a, 2017). The common feature for these stimuli, including speech, is their predictability (Stekelenburg and Vroomen, 2007); the visual cues starting before the sound allow the perceiver to anticipate what is coming and when (Paris et al., 2017). Similarly, prediction is essential in playing music (Koelsch et al., 2019). For example, finger and hand movements in playing the piano will give some prediction about what key will be pressed and when the sound will be started (Sebanz and Knoblich, 2009; Heggli et al., 2019). Thus, the question is, since finger and hand movements start before the audio onset, and provide possible cues for the upcoming musical sound, does AV modulation in music also occur for N1 and P2 amplitudes and latencies, similar to the speech stimuli?

Previous electrophysiological studies on auditory music perception have suggested that N1 and P2 amplitudes, but not latencies, are sensitive to an individual's previous experience, such as musical training (Shahin et al., 2003, 2005; Kuriki et al., 2006; Baumann et al., 2008; Virtala et al., 2014; Maslennikova et al., 2015; Rigoulot et al., 2015; Sanju and Kumar, 2016; but also see Lütkenhöner et al., 2006). Pantev et al. (2001) showed that musicians' early cortical amplitude is higher in response to a piano tone than for non-musicians, and other studies replicate these findings. For example, Shahin et al. (2003) showed that musicians have a more enhanced P2 amplitude in response to music stimuli (piano and violin) than non-musicians. In a later study, they confirmed their results for enhanced P2 amplitude for musicians, compared to non-musicians, in response to different piano tones (Shahin et al., 2005). Other research (e.g., Maslennikova et al., 2015) also have suggested that musicians, compared to non-musicians, have higher amplitude for both N1 and P2 in response to music stimuli.

Musical training as an AV experience also shapes AV perception (Haslinger et al., 2005; Musacchia et al., 2008; Lee and Noppeney, 2011; Paraskevopoulos et al., 2012; Maes et al., 2014; Proverbio et al., 2016). Years of practicing a musical instrument can enhance auditory processing (Pantev et al., 2001; Shahin et al., 2003, 2005; Baumann et al., 2008; Maslennikova et al., 2015) and provide an attractive model for studying experience-based neural plasticity. Years of musical training enrich a musician's multimodal experience and integrate different sensory signals from the auditory, visual, and motor cortex (Zatorre et al., 2007; Strait and Kraus, 2014). For example, one study (Petrini et al., 2009a) suggested that drummers, compared to non-musicians, were more sensitive to AV synchrony in point-light motions of drumming and could perceptually interpolate absent visual information (Petrini et al., 2009b). Moreover, playing an instrument is also a case of auditory-motor association learning. For example, when playing piano, pressing a key to produce a certain pitch will, over time with practice, develop key-to-pitch mapping (Maes et al., 2014). Years of musical training enhance auditory mechanisms related to sub/cortical areas, not only in response to music, such as pitch perception (Kishon-Rabin et al., 2001; Schön et al., 2004; Zatorre et al., 2007; Barnett et al., 2017; Bianchi et al., 2017), but also to other AV events such as speech (Patel, 2011). With this basis, in the present study, the role of previous musical experience will be examined for N1 and P2 in AV music perception during which visual cues from finger and hand movement can offer prediction for the corresponding sound.

While N1 and P2 precisely depict the temporal aspect of neural activity in AV perception, coherence of EEG oscillations is determined by inter-trial phase coherence (ITPC) in response to a stimulus. These EEG oscillations measured by ITPCs particularly in low-frequency (<30 Hz) bands can also shape the generation of evoked potentials, such as N1 and P2 (Gruber et al., 2004; Eggermont, 2007; Edwards et al., 2009; Koerner and Zhang, 2015; van Diepen and Mazaheri, 2018). ITPC in low-frequency bands has previously been used together with ERP analyses to study N1 and P2. For example, Koerner and Zhang (2015) suggested that early evoked potentials such as N1 and P2 might be dependent on ITPC for delta, theta, and alpha-band activities. Moreover, Kühnis et al. (2014) showed that musicians' beta activity increase is accompanied by reduced N1 amplitude in response to a passive vowel listening task. Therefore, in the present study, ITPC will be computed for delta, theta, alpha, and beta to investigate the role of low-frequency bands activity accompanied by N1 and P2.

Many cognitive processes, such as perception, can be linked to synchronized oscillatory networks (Buzsáki and Draguhn, 2004). Notably, low-frequency activity is essential in the processing of speech (Howard and Poeppel, 2012; Gisladottir et al., 2018) and music (Doelling and Poeppel, 2015; Doelling et al., 2019). Low-frequency activity also correlates with early ERP components (Gruber et al., 2004; Fuentemilla et al., 2006; Arnal and Giraud, 2012; Kühnis et al., 2014; Koerner and Zhang, 2015). Moreover, previous research on AV perception in speech, not taking musical experience into account, suggested that visual predictory information signaling an upcoming speech sound might reset ongoing frequency activity (Lakatos et al., 2007; Busch and VanRullen, 2010). With this basis, some illustrative cognitive processes linked to low-frequency oscillations can be mentioned.

Theta activity is reduced in response to AV speech perception (Lange et al., 2013), however, theta sensitivity is not limited to speech perception (Luo and Poeppel, 2012), and is linked to various cognitive functions (Canolty and Knight, 2010), such as syllable level encoding and speech intelligibility (Giraud and Poeppel, 2012; Doelling et al., 2014), as well as multisensory attention (Keller et al., 2017). Theta oscillation also correlates with auditory N1 and P2 amplitude responses to speech syllables (Koerner and Zhang, 2015). Moreover, delta-theta activity is positively correlated with performance in stimulus detection tasks (Arnal and Giraud, 2012). Doelling and Poeppel (2015) proposed that delta-theta activity in response to music stimuli is also correlated with better performance in detection tasks, and corresponding to speech intelligibility, delta-theta activity in response to music may be linked to the identification of individual notes in the sound stream. Moreover, reduced beta and alpha oscillations have been connected to attentional shift (van Ede et al., 2014) and predictory processing (Lange, 2013; Todorovic et al., 2015) to an upcoming stimulus. Alpha oscillation might also be reduced in response to AV speech, which might be connected to selective attention mechanisms (Foxe and Snyder, 2011; Lange, 2013), as well as mechanisms regulating attention and inhibition (Strauß et al., 2014). In addition, beta-band activity supports auditory-motor interactions and encoding of the musical beat (Large and Snyder, 2009). Beta-band activity has an essential role in predictive timing (Arnal and Giraud, 2012; Doelling and Poeppel, 2015) and in cognitive functions, especially in tasks that require top-down control procedures (Engel and Fries, 2010). Additionally, the beta oscillation is linked to the phase of delta activity in sensory-motor areas (Cravo et al., 2011). As AV modulation at N1 and P2 may coincide with ITPC fluctuations in response to AV speech stimuli, especially for theta activity (e.g., Edwards et al., 2009), a reduction in N1 and P2 amplitudes is expected to correspond to lower ITPC values. Therefore, ITPCs in low-frequency bands delta, theta, alpha, and beta are expected to be lower in AV music perception compared to the perception of auditory music.

In response to auditory music, previous research has shown that musicians, relative to non-musicians, have higher ITPC values in delta and theta bands, which was correlated to their years of training and perceptual accuracy (Doelling and Poeppel, 2015). Musicians also showed higher delta activity in response to music stimuli compared to non-musicians (Bhattacharya and Petsche, 2005). Some studies (Trainor et al., 2009; Bidelman et al., 2014; Bidelman, 2017) argue that musical experience may also regulate oscillatory activity, such as alpha and beta, in response to speech and non-speech stimuli. Playing a musical instrument involves sensory-motor practice (Zatorre et al., 2007), which can regulate beta activity (Fujioka and Ross, 2017). Consequently, ITPC for musicians and non-musicians in AV music will be studied to examine whether musicians, compared with non-musicians, show greater ITPC values in auditory and lower ITPC values in AV music perception along with their N1 and P2 amplitudes in auditory and AV music perception.

In sum, in the current study, musicians and non-musicians are first compared based on their ERP and ITPC responses to auditory music, and based on prior findings (e.g., see Shahin et al., 2005; Baumann et al., 2008), N1 and P2 amplitudes for musicians are expected to be relatively enhanced in auditory perception, compared to non-musicians. Next, auditory and AV music are compared between groups based on N1 and P2 amplitudes and latencies to examine the effect of the potentially predictive visual cues from a musical instrument being played starting before the upcoming musical sound. Both groups are expected to show lower amplitudes and latencies with AV music compared with auditory music (e.g., Stekelenburg and Vroomen, 2007).

A novel contribution of the current study is its inclusion of time-frequency analyses of trial-by-trial fluctuations in delta (0.5–4 Hz), theta (4–8 Hz), alpha (8–12 Hz), and beta (12–30 Hz) in response to auditory and AV music stimuli. As low-frequency oscillations correlate with early ERP components (e.g., Koerner and Zhang, 2015), ITPCs for delta, theta, alpha and, beta-band in auditory perception are expected to be higher for musicians than for non-musicians, and for both groups to be lower for AV music than auditory music. Furthermore, as previous research (e.g., Petrini et al., 2009a,b) suggested that relative to non-musicians, musicians have enhanced AV perception, musicians in the current study are hence expected to have lower N1 and P2 amplitudes and latencies, as well as lower ITPC values in corresponding frequency bands.

## 2. Materials and Methods

This experiment was designed to investigate the effect of musical experience on auditory music perception by comparing musicians and non-musicians, as well as the effect of visual information from hand and finger movements predicting the upcoming sound in AV music. For both aspects of the study, data were based on N1 and P2 amplitudes and latencies and corresponding ITPC values. Data collection reported here for AV music perception were recorded within a larger study on AV perception (see, e.g., Sorati and Behne, 2019).

### 2.1. Participants

As shown in Table 1, 41 participants (aged between 19 and 33 yrs) were among the students at the Norwegian University of Science and Technology (NTNU), among which 20 were musicians and 21 were non-musicians. For technical reasons, data from one musician were removed from the study. All participants had Norwegian as a first language, were right-handed based on a variant of the Edinburgh Handedness Inventory (Oldfield, 1971), had normal hearing (15dB HL pure tone auditory threshold for 250–4,000 Hz, British Society of Audiology, 2004), and normal to corrected visual acuity (Snellen test). None of the participants had a history of neuropsychological disorders.

**Table 1:** The descriptive information, means, and standard deviations in the parentheses, for musicians and non-musicians based on participant information.

	**Age**	**Gender**	**Interest in music**	**Listening to music per week**	**Age of starting an instrument**	**Musical experience**	**Hours of practice per week**
Musicians	23 yrs (3 yrs)	10 females, 9 males	9(1) / 10	19 h (13 h)	8 yrs (2 yrs)	14 yrs (3 yrs)	15 h (11 h)
Non-musicians	23 yrs (3 yrs)	10 females, 11 males	5(2) / 10	5 h (5 h)	-	Less than a year	-

Musicians were students at NTNU in Music Performance Studies or Musicology, where admission requirements include evaluation of music theory and performance as well as advanced instrumental skills. None of the musicians had absolute pitch perception. In the timeframe of the experiment, musicians were actively playing an instrument and were regularly performing in public. Musicians had expertise with instruments including piano, keyboard, guitar, percussion, violin, and saxophone. As general properties of musical training rather than specific practice with temporal cues are sufficient for cognitive enhancement, variation in musical instruments played by musicians is not expected to affect the results (Pantev et al., 2001; Kühnis et al., 2013). However, since other research (Pantev et al., 2001; Heggli et al., 2019) suggest that the effect of musical expertise is specific for each musical instrument, here, all musicians had keyboard or piano as their main or secondary instrument. The formal musical training for musicians started at a mean age of 8 years and had been playing their main instrument for at least 8 years. Participants' interest in music was measured based on a self-reported scale from (1 = “not interesting at all”) to 10 (“very interesting”), and musicians reported, on average, 9. Musicians with dancing and singing experience were excluded from this study, to isolate the effect of musical training to instrumentalists.

Non-musicians were also NTNU students, although not in music, and had no more than the once per week music training for 1 year which is obligatory in Norwegian elementary schools. The non-musicians' self-reported their interest in music was, on average, 5 on the 10-point scale.

All participants signed the consent form registered with the Norwegian Center for Research Data, and received an *honorarium* for their participation in the experiment.

### 2.2. Stimuli

AV materials were recorded in an IAC sound-attenuated studio (IAC acoustics, Hampshire, UK) at the NTNU Speech Laboratory, Department of Psychology, NTNU. A Sony PMW-EX1R camera (30 fps) connected to an external Røde NT1-A microphone (Sydney, Australia), mounted on a tripod was used to video record an instrumentalist's right hand positioned on a keyboard (Evolution MK-449C, UK) and with the left side of the right thumb depressing the middle C4 (261.6 Hz) key and the tips of other fingers resting on the next four white keys (i.e., D4, E4, F4, and G4). This position allowed clear visibility of the finger and hand movements while depressing the key. Using Adobe Premiere Pro CS54.5, the audio from the video recording was replaced with a pure MIDI C4 produced in GarageBand (10.0.3). Videos were then exported in H.264 format with an MP4 container.

**Figure 1:**
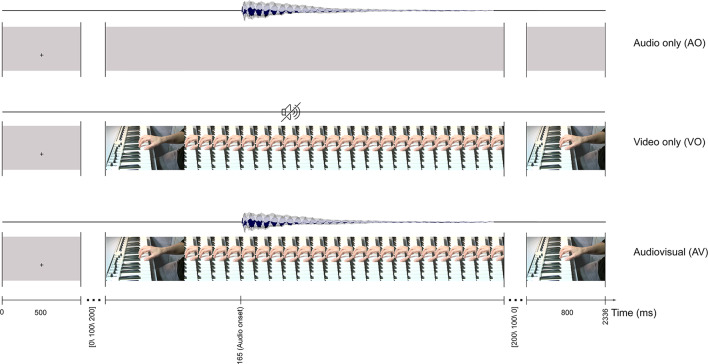
Timeliness for the audio only, video only, and audiovisual stimuli.

As shown in Figure 1, these materials were the basis for three sets of music stimuli: audio only (AO), in which the 700 ms-long audio signal was presented with a visual gray background; the video only (VO), which was the original video recording from the finger and hand movement with no sound; and the audiovisual (AV), in which the synchronized audio and video recordings were presented. In addition to these, a stimulus with a gray background and no audio was included in the experiment, but is not directly relevant for the issues addressed here, and is not addressed below.

### 2.3. Procedure

The experiment was conducted in a dimly lit IAC sound-attenuated studio at the NTNU Speech Laboratory. Participants used a chinrest to maintain a stable head position and generally reduce movements. The visual stimuli were presented on a 40" LCD flat panel display (Samsung SyncMaster 400DX-2) with a 1152 × 648 resolution, positioned at eye level 190 cm in front of the participant. The video size and position were chosen to correspond to the actual size of a MIDI keyboard. Audio was played over ER1-14B insert earphones via an HB7 headphone buffer (Tucker-Davis Technologies, US). The sound pressure level for the audio stimuli on average was 65 dB, based on measurement with a NOR140 digital sound level meter (Norsonic, Norway).

The audio and video presentation delays when playing the stimuli were recorded for AO, VO, and AV stimuli using an audiovisual delay test toolbox (Electrical Geodesics, Oregon, US), connected to the EEG system (Electrical Geodesics, Oregon, US). Presentation delays for audio (50 ± 12 ms jitter), and video (57 ± 2 ms jitter) were compensated in the analysis.

Prior to the experiment, each participant was instructed to limit eye movements, and to try to relax. Participants were informed that their task was to detect target trials and press a button on a Response Pad 200 (Electrical Geodesics, USA). The target trials were included to engage the participants in the task (ca. 10% of the trials). As previous research (Wild et al., 2012) has shown that attentional influences on the sensory processing are related to the modality of the stimulus, targets in the target trials were the same modality as non-target trials. Specifically, in the AO target trials a 120 ms-beep was presented with two onset variations: one beginning 200 ms and another one 400 ms after the audio onset. In the VO target trials a 120 ms-white dot was presented above or below the C4 keyboard key. In the AV target trials a white dot was synchronized with a tone (500 Hz, 120 ms). After receiving instructions about the experimental task, participants completed 5 practice trials to ensure they understood the experimental task.

As presented in Figure 1, a trial started with a fixation cross on a gray background (500 ms), presented at the position of the C4 key. The fixation cross was followed by a still keyboard image with a random interval of [0, 100, 200] ms until the video started. The first detectable finger movement frame (video onset) started 165 ms preceding the auditory onset. Each AV stimulus lasted 1,036 ms (31 frames), and the last frame of the video was displayed (800 ms).

The experiment took about 50 minutes, presenting 738 pseudo-randomized trials in four blocks, including 72 target trials and 246 trials for each stimulus. The breaks between the blocks were 3-minute long and each block contained eight short pauses.

### 2.4. EEG Recordings

Raw EEG data were recorded at a 1,000 Hz sampling rate with a 128-channel dense array EEG system cabled to a Net Amps 300 amplifier (Electrical Geodesics, Oregon, US). Psychtoolbox (Pelli and Vision, 1997) was used for stimulus presentation, and EEG was recorded with Net Station (5.2.0.2). During the experiment the experimenter used a separate display to monitor stimuli and EEG channels. No online filters were applied, and Cz was used as the default reference. Before the session started for each participant, a cap was selected based on head size measured from the nasion to the inion and the left-right preauricular distance. To improve the electrode-to-scalp conduction, participants were asked to brush their hair (Luck, 2014) prior to the cap being applied with Cz positioned at the midpoint of the nasion. Impedances were maintained below 100 *K*Ω.

### 2.5. Data Analyses

#### 2.5.1. Preprocessing

EEG recordings were interpolated to the 10-20 system (Jasper, 1958) and imported into Matlab R2015b with the EEGLAB (v15) extension (Delorme and Makeig, 2004) which was used for the full analysis. In EEGLAB, a high-pass filter (0.5, 12 dB/octave) and a low-pass filter (48 Hz, 12 dB/octave) were applied to the raw continuous data. After removing bad channels, the remaining channels were re-referenced to the average reference. Large artifacts, such as head movements, were later removed from the data. Independent component analysis was performed to remove the stereotypical artifacts (e.g., eye blinks).

#### 2.5.2. Event-Related Potential

Preprocessed EEG data were epoched from 200 ms before, to 500 ms after the audio stimulus onsets (i.e., each epoch was 700 ms), and −200 ms to 0 ms was selected for baseline correction. N1 was defined in a window of 75–125 ms and P2 in a window of 175–225 ms. Since Cz displays activity from the auditory brain areas (Bosnyak et al., 2004), and has been previously used in the field (e.g., Van Wassenhove et al., 2005), Cz was therefore chosen for further analyses. Separately for each participant, and based on the non-target trials, average ERPs were calculated for each condition (AO, VO, AV).

Musicians have been shown to have enhanced N1 and P2 amplitudes (Shahin et al., 2003, 2005; Baumann et al., 2008; Maslennikova et al., 2015). Therefore, first the difference between musicians and non-musicians for N1 and P2 in the AO condition was investigated. Then, a difference wave (AV-VO) was calculated by subtracting VO signals from the AV signals, to extract the contribution of the visual waveform from the AV ERPs. Furthermore, to examine N1 and P2 amplitude and latency reduction due to the predictive visual cues for a coming audio signal, for each group N1 and P2 from AV-VO were compared with N1 and P2 from the AO condition (AO vs. AV-VO) (Van Wassenhove et al., 2005; Baart, 2016). Finally, musicians and non-musicians were compared based on their N1 and P2 amplitudes and latencies.

In addition, to investigate spatio-temporal activity of AV modulation (in anterior, posterior and lateral sites), separately for musicians and non-musicians, AO and AV-VO were compared by applying pointwise *t*-tests in a window of 1–250 ms for 8 electrodes (F3, Fz, F4, C3,C4, P3, Pz, P4) in addition to Cz.

#### 2.5.3. Inter-trial Phase Coherence

ITPC is a measure of the phase synchrony across trials as a function of frequency in the epoch time series and time point, which can be computed for different frequencies. ITPC values are defined between one and zero. While one suggests total phase coherence, zero value suggests arbitrary phase distribution across trials (Cohen, 2014).

(1) $$\begin{array}{r} ITPC_{tf}=|n^{-1}\overset{n}{\underset{r=1}{\sum }}e^{ik_{tfr}}| \end{array}$$

In Equation 1 *t*, *f*, and *n* stand for time, frequency, and the number of trials, respectively, and *e*^*ik*^ is the Fourier transform index at *t* and *f*.

Preprocessed EEG data were segmented into 2,400 ms epochs, from 1,200 ms before to 1,200 ms after the auditory onset. ITPC 1 was run with EEGLAB toolbox function “newtimef” (Delorme and Makeig, 2004) in a window of 75–225 ms which matches N1 and P2 latencies, for low-frequency bands (<30 Hz), such as delta (0.5–4 Hz), theta (4–8 Hz), alpha (8–12 Hz), and beta (12–30 Hz).

#### 2.5.4. Statistical Analyses

Repeated-measures analyses of variance (ANOVA), with α = 0.05, were performed with SPSS (v. 25) to assess condition (AO vs. AV-VO) and musical experience (musicians vs. non-musicians) as well as their interactions, both for N1 and P2 amplitudes and latencies at electrode Cz. Since N1 and P2 have previously been shown (e.g., Arnal et al., 2009) to rely on different mechanisms for predictory visual cues in AV perception, an interaction between N1 and P2 was not expected, and they were therefore analyzed separately. Similarly, ANOVAs were run for ITPC in the delta, theta, alpha, and beta-band activity. In the two-way ANOVA analyses both for ERP and ITPC, a main effect of experience would collapse data from AO and AV-VO conditions and would not give a meaningful comparison between musicians and non-musicians. Therefore, for a precise comparison between the two groups, musicians and non-musicians, were compared in AO music perception, and in the interaction between condition and musical experience. While the main effect of experience is not directly addressed, *F*-values are presented in Table 4 for evaluation.

For EEG, factors affecting the signal-to-noise ratio in a condition (e.g., noise, number of trials) have consequences for statistical reliability, yet are not commonly reported (Cohen, 2014). For instance, even with environmental and systemic noise at a minimum, for statistical reliability, Luck (2014) suggested using a set number of trials for specific ERP components. For ITPC analyses, the number of trials in a condition can be used to calculate the strength of the ITPC (Cohen, 2014), and to achieve this in the current study, a bootstrapping algorithm was run for each frequency band between 75 and 225 ms, corresponding to the N1 and P2 windows. First, to run the convolution over the signal, a Gaussian function centered for each frequency band (for delta at 2.5 Hz, theta at 6 Hz, alpha at 10 Hz, and beta at 21 Hz) was used as a wavelet function. The average ITPC for the convoluted signal in a time window was calculated with the bootstrap algorithm iterated 50 times for each trial. Statistical analyses were carried out (*p* < 0.01) for the AO and AV conditions for musicians and non-musicians. The maximum number of trials needed for the ITPC analyses among all groups and conditions was considered the minimum threshold (n = 501) for each condition and group.

## 3. Results

As detailed in Table 2, on average across conditions, musicians correctly responded to 95% and non-musicians to 94% of the target trials with similar standard deviations, indicating that the groups had a comparable focus on the stimuli during the experiment.

**Table 2:** Mean percentages, with standard deviations in parentheses, for correct responses to target trials in the audio only, video only, and audiovisual conditions.

	**Audio only condition**	**Video only condition**	**Audio visual condition**	**Average**
Musicians	95% (1)	95% (1)	96% (1)	95% (2)
Non-musicians	93% (1)	95% (1)	94% (1)	94% (3)

### 3.1. Audio Only Condition

**Figure 2:**
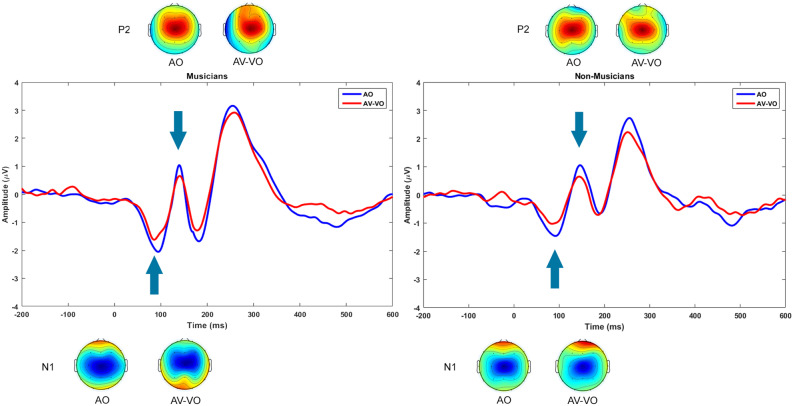
Grand averaged event-related potentials at Cz and topographical maps for N1 and P2, plotted for audio only (blue) and audiovisual minus video only (red).

**Figure 3:**
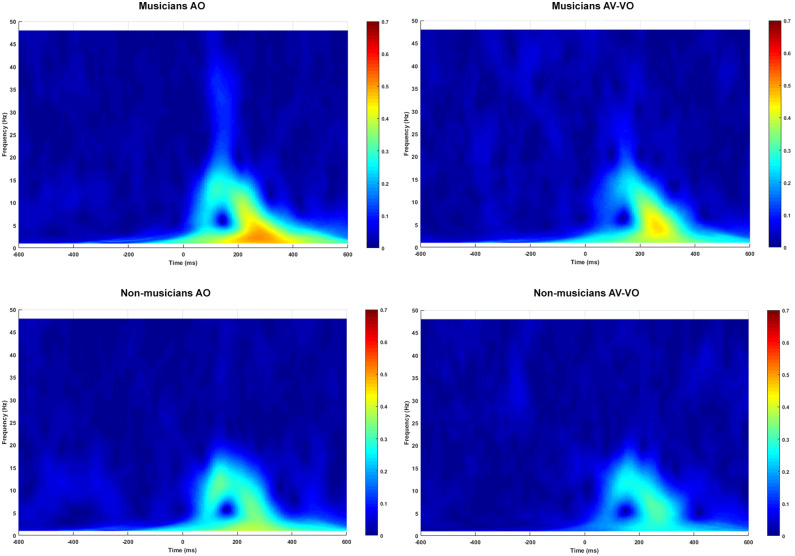
Musicians and non-musicians' ITPC spectrum (0.5–48 HZ) for audio only (AO) and audiovisual minus video only (AV-VO).

As summarized in Table 3 and shown in Figure 2, for the AO condition, musicians and non-musicians were compared for N1 and P2 amplitudes and latencies, as well as for their trial-by-trial phase coherence, as is shown in Figure 3.

**Table 3:** ERP and ITPC means and standard deviations (in the parentheses) for musicians and non-musicians in audio only (AO) and audiovisual minus video only (AV-VO).

		**Event-related potential (ERP)**				**Inter-trial phase coherence (ITPC)**			
		**N1**		**P2**					
		**Amplitude (μ*V*)**	**Latency (ms)**	**Amplitude (μ*V*)**	**Latency (ms)**	**Delta**	**Theta**	**Alpha**	**Beta**
Musicians	AO	−2.20 (0.60)	96 (7)	1.48 (1.5)	142 (7)	0.40 (0.07)	0.36 (0.09)	0.36 (0.09)	0.24 (0.07)
	AV-VO	−1.86 (0.87)	90 (12)	0.86 (0.86)	140 (5)	0.36 (0.08)	0.30 (0.09)	0.31 (0.09)	0.19 (0.04)
Non-musicians	AO	−1.65 (0.88)	93 (14)	1.38 (0.74)	147 (10)	0.31 (0.10)	0.33 (0.09)	0.39 (0.11)	0.18 (0.04)
	AV-VO	−1.27 (0.77)	83 (15)	0.98 (0.89)	145 (12)	0.26 (0.11)	0.27 (0.08)	0.33 (0.12)	0.18 (0.06)

#### 3.1.1. Event-Related Potentials

For the AO music condition, one-way analyses of variance (ANOVA) were carried out comparing musicians and non-musicians for N1 and P2 amplitudes and latencies. Results in Table 3 show that musicians had a higher N1 amplitude (Δ = 0.55μ*V*) than non-musicians [*F*_(1,38)_ = 5.25, *p* = 0.2], whereas N1 latency [*F*_(1,38)_ = 0.62, *p* = 0.43], P2 amplitude [*F*_(1,38)_ = 0.07, *p* = 0.77], and P2 latency [*F*_(1,38)_ = 3.2, *p* = 0.08] showed no significant difference between the two groups.

#### 3.1.2. Inter-trial Phase Coherence

For the AO music condition one-way ANOVAs comparing musicians and non-musicians were run for the delta, theta, alpha, and beta bands. As summarized in Table 3, results showed a significant enhancement of delta-band activity for musicians compared to non-musicians [*F*_(1,38)_ = 6.54, *p* = 0.01]. Furthermore, while theta [*F*_(1,38)_ = 0.92, *p* = 0.34] and alpha [*F*_(1,38)_ = 1.03, *p* = 0.31] showed no significance difference between the two groups, beta activity was significantly [*F*_(1,38)_ = 6.94, *p* = 0.01] higher for musicians than for non-musicians.

### 3.2. Audiovisual Modulation

The AO condition was linked with the audio from the corresponding AV (i.e., AV-VO), and musicians and non-musicians were then compared based on condition (AO vs. AV-VO) for amplitudes and latencies of N1 and P2 (Figure 2), likewise for ITPC in each of the frequency bands (Figure 3). Means are shown in Table 3, and F-statistics are presented in Table 4.

**Table 4:** Summary of F-statistics for ERP and ITPC data.

	**Event-related potential (ERP)**				**Inter-trial phase coherence (ITPC)**			
	**N1**		**P2**					
	**Amplitude**	**Latency**	**Amplitude**	**Latency**	**Delta**	**Theta**	**Alpha**	**Beta**
Condition (AO vs. AV-VO)	11.15^*^	11^*^	19.82^**^	2.45	11.55^*^	23.62^**^	12.79^*^	8.78^*^
Experience (musicians vs. non-musicians)	10^*^	7.52^*^	0.001	5.10^*^	18.28^**^	1.81	1.40	5.63^*^
condition × Experience	0.01	0.55	0.98	0.19	0.03	0.00	0.10	5.48^*^

* p ≤ 0.05,

** *p < 0.0001*.

#### 3.2.1. Event-Related Potentials

Repeated-measures ANOVA was carried out to investigate the effect of condition (AO vs. AV-VO) and its interaction with participants' musical experience (musicians vs. non-musicians) for amplitudes and latencies of N1 and P2 components. Results from the main effect of condition for N1 amplitude [*F*_(1,38)_ = 11.15, *p* = 0.002], N1 latency, [*F*_(1,38)_ = 11, *p* = 0.002], and for P2 amplitude [*F*_(1,38)_ = 19.82, *p* = 0.00007] showed a smaller N1 amplitude and latency and P2 amplitude in AV-VO compared to the AO condition. However, the ANOVA for P2 latency [*F*_(1,38)_ = 2.45, *p* = 0.12] showed no significant difference between the two conditions.

Results showed no significant interaction between condition and experience for N1 amplitude [*F*_(1,38)_ = 0.01, *p* = 0.89], N1 latency [*F*_(1,38)_ = 0.55, *p* = 0.46], P2 amplitude [*F*_(1,38)_ = 0.98, *p* = 0.32] or P2 latency [*F*_(1,38)_ = 0.19, *p* = 0.66]. As described above, in the AO condition the N1 amplitude was enhanced in musicians compared to non-musicians, which might have contributed to the nonsignificant interaction for N1 amplitude. A one-way analysis of covariance was therefore conducted for AV-VO comparing N1 amplitude in musicians and non-musicians with AO N1 amplitude as a covariate. Results showed no significant difference between musicians and non-musicians' AV-VO N1 amplitude after controlling for AO for N1 amplitude [*F*_(1,38)_ = 0.87, *p* = 0.35].

**Figure 4:**
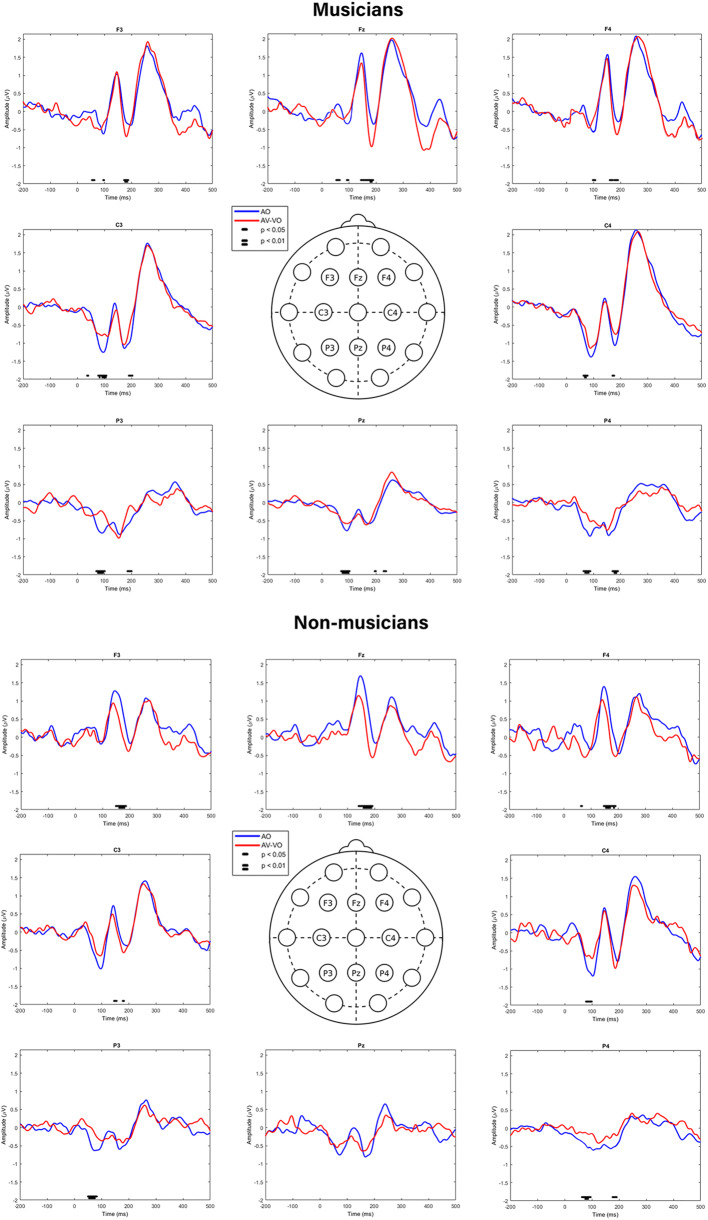
Pointwise two-tailed *t*-test comparison of AO and AV-VO ERPs at F3, Fz, F4, C3, C4, P3, Pz, P4.

In addition, a pointwise two-tailed *t*-tests were used to examine the difference between ERPs for AO and AV-VO at F3, Fz, F4, C3, C4, P3, Pz, P4, for musicians, and for non-musicians. As illustrated in Figure 4, results showed that in the frontal sites (F3, Fz, and F4) for both groups, AV-VO is lower around 100 ms and 200 ms after the stimuli onset, compared to the AO waveform. However, in lateral and posterior sites (C3, C4, P3, Pz, P4), while for musicians AV-VO was lower than the AO condition, for non-musicians this modulation pattern was less evident.

#### 3.2.2. Inter-trial Phase Coherence

For ITPC in each of the frequency bands, ANOVAs were carried out to examine the main effect of condition and its interaction with musical experience. As shown in Table 3, results for condition showed significantly lower ITPC values in AV-VO compared to the AO condition for delta [*F*_(1,38)_ = 11.55, *p* = 0.002], theta [*F*_(1,38)_ = 23.62, *p* = 0.00002], alpha [*F*_(1,38)_ = 12.79, *p* = 0.001] and beta [*F*_(1,38)_ = 8.78, *p* = 0.005].

Whereas the interaction between condition and experience was not significant for delta [*F*_(1,38)_ = 0.03, *p* = 0.85], theta [*F*_(1,38)_ = 0, *p* = 0.99] or alpha [*F*_(1,38)_ = 0.1, *p* = 0.75], the interaction was significant for ITPC in the beta-band [*F*_(1,38)_ = 5.48*p* = 0.02]. One-way repeated-measures ANOVAs with multiple comparison Bonferroni correction showed that ITPC in the beta-band for musicians was significantly lower for AV-VO compared to the AO condition [*F*_(1,18)_ = 11.41, *p* < 0.025], while results for non-musicians did not show this pattern [*F*_(1,20)_ = 0.24, *p* = 0.62].

In summary, in response to auditory music stimuli, musicians showed a higher N1 amplitude as well as higher delta and beta ITPCs compared to non-musicians. In AV music perception, when the visual cues predict the upcoming sound, both groups had lower N1 amplitude and latency, and P2 amplitude for AV-VO compared to the AO condition. While both groups showed lower delta, theta, and alpha ITPCs in response to AV-VO music compared to auditory music, only musicians had a lower beta ITPC in AV-VO relative to the AO condition.

## 4. Discussion

To position the current study with previous research, musicians and non-musicians were first compared in auditory music perception based on their ERPs and ITPCs. The current study then extended previous findings by studying whether the AV modulation from visual and hand movements is predicting an upcoming piano sound is affected by musical experience. Consequently, musicians and non-musicians were compared based on N1 and P2, as and ITPC values in the delta, theta, alpha, and beta frequency bands.

### 4.1. Auditory Music Perception

Previous research has shown that musicians have enhanced auditory music perception (Pantev et al., 2001; Shahin et al., 2003, 2005; Kuriki et al., 2006; Baumann et al., 2008; Virtala et al., 2014; Maslennikova et al., 2015; Rigoulot et al., 2015, for a review see, Sanju and Kumar, 2016, but also see, Lütkenhöner et al., 2006). Electrophysiological studies show that musicians, compared to non-musicians, demonstrate higher amplitudes within 100 ms after the onset of musical sound (e.g., Pantev et al., 2001; Baumann et al., 2008; Rigoulot et al., 2015). For example, Baumann et al. (2008) showed auditory music stimuli elicited a higher N1 amplitude, but not P2 amplitude, for musicians compared to non-musicians. In contrast, Shahin et al. (2003) have shown that musicians, relative to non-musicians, elicited a higher P2 amplitude in response to auditory music stimuli. Likewise, other studies (Shahin et al., 2005; Maslennikova et al., 2015) also showed that musicians have higher N1 as well as P2 amplitudes than non-musicians. Consistent with these previous findings (Baumann et al., 2008), auditory music in the current study evoked an enhanced N1 amplitude for musicians compared to non-musicians, and while the musicians' mean P2 amplitude was slightly higher than for the non-musicians (Shahin et al., 2003, 2005; Maslennikova et al., 2015), this group difference was not significant (Baumann et al., 2008).

Although in the current findings, the average P2 amplitude is higher for musicians than non-musicians, the musicians' greater variability in P2 amplitude (Table 3) might be behind the musicians not showing a significantly higher P2 amplitude than non-musicians. In addition to the previous musical experience, top-down attentional processes are a source of variation which might influence the P2 component both for musicians and non-musicians, while, N1 amplitude is less sensitive to attentional processes which lead to less variation (Baumann et al., 2008) and consequently significant difference between musicians and non-musicians due to previous musical experience. Moreover, research showed variation in N1 and P2 might be due to factors other than musical experiences, such as experimental task (Näätänen et al., 2011), and individual differences (Liem et al., 2012; Tan et al., 2016). Although previous research (e.g., Shahin et al., 2003, 2005; Baumann et al., 2008) does not unequivocally show N1 and P2 amplitude enhancement for musicians in response to music stimuli, compared to non-musicians, research does generally show that musicians have enhanced amplitude at N1 or P2 or both of the components. Therefore, Baumann et al. (2008) suggested that in lieu of assigning distinct roles to the enhanced N1 and P2 due to musical experience, these components could rather be considered as an enhanced N1-P2 complex.

As generation of N1 and P2 are superimposed on the trial-by-trial phase alignments, ITPCs for auditory music perception were examined, with findings that ITPC for beta and delta oscillations are enhanced for musicians compared to non-musicians. These findings are consistent with previous research (Bhattacharya and Petsche, 2005) showing that, while listening to music, synchrony in the delta-band is enhanced for musicians compared to non-musicians, as well as similar studies showing that musicians have enhanced beta-activity while listening to music compared to non-musicians (Trainor et al., 2009; Doelling and Poeppel, 2015).

### 4.2. Audiovisual Modulation in Music Perception

The current study evaluated whether musicians and non-musicians differ in how the predictive visual cues from a musical instrument being played affect perception of an upcoming musical sound. Previous research (e.g., Baart, 2016) showed that adding visual information to the auditory signal evokes lower amplitudes and latencies for N1 and P2, and consequently, ITPC values would be expected to decrease as well (Edwards et al., 2009). Baart (2016), based on twenty studies with speech stimuli, suggested that the size of the N1 and P2 amplitude and latency suppression in AV perception might be positively correlated with the amplitudes and latencies of the auditory condition, suggesting a relatively direct relation between early ERPs in auditory and AV perception. This proposal can be extended to the observed findings with musical experience. While musicians in the auditory music condition had a higher N1 amplitude and higher ITPC values in the delta and beta-bands than non-musicians, a corresponding difference could be expected with AV music perception. Moreover, sensory-evoked potentials, such as N1 and P2, and ITPCs in low-frequency activities co-exist for all cortical processing (Koerner and Zhang, 2015; van Diepen and Mazaheri, 2018), and low-frequency (<30 Hz) phase-resetting is likely to contribute to the generation of cognitive ERP components (Edwards et al., 2009; Schroeder and Lakatos, 2009). Therefore, in AV music perception ITPCs in low-frequency bands could also be expected to be suppressed along with N1 and P2, although nevertheless suppressed more for musicians than non-musicians (e.g., Panasiti et al., 2016).

#### 4.2.1. Event Related Potentials

The current study evaluated amplitudes and latencies of N1 and P2 in the perception of auditory music compared with the auditory component of AV music. Previous research suggested that visual predictory cues starting before the auditory signal modulate AV perception, leading to lower amplitudes and latencies of N1 and P2 both with speech (Van Wassenhove et al., 2005; Stekelenburg and Vroomen, 2007; Arnal et al., 2009; Paris et al., 2016b) and non-speech stimuli (Stekelenburg and Vroomen, 2007; Vroomen and Stekelenburg, 2010; Paris et al., 2016a, 2017). Consistent with previous research, in the current study, both groups showed a lower N1 amplitude and latency and P2 amplitude for AV music compared to auditory music perception.

Although both groups showed an earlier P2 peak for AV music compared to auditory music, this difference was not significant. In line with the current results, Paris et al. (2017) did not report any decrease for P2 latency with non-speech-stimuli. In a meta-analysis of AV speech perception, Baart (2016) showed that, although most of the studies show a smaller P2 latency in AV compared to the auditory speech, having no decrease of latency for P2 is not uncommon (e.g., Pilling, 2009; Baart et al., 2014). Furthermore, in contrast with the current study's results, Stekelenburg and Vroomen (2007) showed a decrease for P2 latency for non-speech stimuli. Therefore, although P2 latency is generally lower in an AV vs. an auditory condition, a lack of a lower P2 latency in AV perception also has been reported in response to speech and non-speech stimuli.

Previous research (e.g., Stekelenburg and Vroomen, 2007; van Wassenhove, 2013) suggested that AV modulation due to visual cues predicting the upcoming sound on early evoked potentials is mostly visible at central sites. For example, Stekelenburg and Vroomen (2007) showed that AV modulation which leads to lower amplitudes and latencies for N1 and P2 in AV perception compared to the auditory condition, is more evident in fronto-central electrodes for non-speech stimuli, compared to the speech stimuli. Similarly, the current findings suggested that for both groups, the AV modulation effect is strongest in fronto-central sites around 100 ms, and 200 ms after stimulus onset. However, while musicians also showed AV modulation bilaterally and posteriorly, for non-musicians AV modulation was less evident at posterior sites, such as Pz. These findings are in line with fMRI research (Petrini et al., 2011) suggesting that musicians compared to non-musicians showed lower neural activity bilaterally and in more posterior areas, such as the cerebellum, in response to an AV simultaneity judgment task. Together, these findings suggest that while AV modulations on early evoked potentials are generally more evident at fronto-central electrodes, musicians, compared to non-musicians, also show such modulations in posterior areas of the brain.

#### 4.2.2. Inter-trial Phase Coherence

ITPCs in low-frequency bands are likely to shape the generation of N1 and P2 (e.g., Edwards et al., 2009) and co-exist with these components for all cortical processing (e.g., Koerner and Zhang, 2015). Therefore, here, for AV music relative to auditory music, lower N1, and P2 amplitudes are expected to coincide with lower ITPC values for delta, theta, alpha, and beta. Findings showed lower N1 amplitude and latency, and P2 amplitude, together with lower ITPCs in delta, theta, and alpha-band activity in AV music perception, relative to the auditory music perception condition. Nevertheless, while musicians showed lower beta-band activity in AV perception, relative to the auditory music perception, non-musicians did not show such suppression.

These results are also consistent with previous research on visual cues predicting the upcoming speech sound in AV speech perception. For example, Arnal et al. (2011) suggested that theta oscillation is decreased in response to AV speech perception. Theta and delta oscillations reflect visual predictiveness of the stimuli (Arnal and Giraud, 2012), and also, signal the processing of correctly predicted stimuli. The predictiveness of the visual cues also modulates the phase in delta-theta activity, which can provide an explanation for the cross-modal benefits of visual cues in AV speech perception studies (Arnal et al., 2011). Furthermore, as with the current findings, Stefanics et al. (2010) showed a decrease in earlier processing of delta-band activity in response to correctly predicted stimuli. Along with expected findings for ITPCs in the theta and delta-bands, current results further indicate that ITPC for both groups showed desynchronization for the alpha-band in AV compared to auditory music perception. Other studies also suggest that the onset of the predictory visual cues itself can lead to substantial lower amplitude of ongoing alpha activity (Foxe and Snyder, 2011; Arnal and Giraud, 2012) which is consistent with research on anticipatory attention (Bastiaansen and Brunia, 2001; Bastiaansen et al., 2001) with speech (Arnal and Giraud, 2012; Gisladottir et al., 2018), and tactile stimuli (van Ede et al., 2014). In summary, findings in the current study show that coinciding with N1 and P2 amplitudes, ITPCs in delta, theta, and alpha are lower in AV music due to visual cues predicting the upcoming musical sound, compared to the auditory music, regardless of musical experience.

In the current study, ITPC for beta activity showed a different pattern of results than for the other frequency bands studied; for non-musicians, the ITPC for the beta-band showed no difference between AV and auditory music perception, whereas for musicians the ITPC for the beta-band was lower in AV music perception compared to the auditory condition. Beta activity is widely associated with motor tasks, the response time (Senkowski et al., 2005), and coordination within the sensory-motor system (Baker, 2007; Lalo et al., 2007). Previous research showed that beta oscillations first decrease and then increase synchronized with the musical beat of stimuli (Fujioka et al., 2009, 2015). Fujioka et al. (2012) also suggested that beta oscillation shows a similar decrease and rebound to temporal anticipation during the beat perception, which engages motor-related areas despite no intended movement. Moreover, previous research on AV speech suggested that beta oscillations are associated with accuracy in the temporal prediction of the upcoming stimuli (Arnal et al., 2015) as well as feedback loops and prediction errors (Arnal et al., 2011; Arnal, 2012; Arnal and Giraud, 2012). For example, beta activity is higher in response to incongruent AV stimuli than congruent stimuli (Paris et al., 2016b). In another study, Fujioka et al. (2009) showed that a beta decrease, which usually occurs after a regular musical beat, was absent after the omission of an expected beat. Furthermore, previous research (Fujioka and Ross, 2017) also showed that, after 1 month of musical training, beta activity increases during passive listening to a metronome for older adults. Moreover, based on topographical analysis, Mikutta et al. (2014) suggested that musicians, compared to amateurs, have enhanced beta activity (between 19 and 23 Hz) in central cortical areas in response to emotional music. Together, these studies suggest that in AV perception, the low ITPC values in the beta-band for musicians can be a consequence of their musical training with the use of visual information to temporally predict an upcoming sound.

In short, the proposal by Baart (2016) that findings for AV perception may be correlated with those for auditory perception is generally supported by the ERP and ITPC results for music in the current study. However, findings here, in addition, showed notable differences between musicians and non-musicians and highlight experience as a factor influencing AV perception.

### 4.3. Musicians and Sensory-Motor Systems

To isolate the neuroplastic effect of musical training to instrumental music, for the current study the musician group consisted of highly trained instrumentalists, a form of training which excludes vocal and dance training which might lead to functional and structural differences compared to instrumental training, and thereby affect auditory perception (Halwani et al., 2011; Poikonen et al., 2018). For instance, sensory-motor and pre-motor cortices, as well as the superior temporal sulcus, have been shown to structurally differ for dancers and non-dancers (Hänggi et al., 2010). Moreover, beta oscillation in the auditory cortex facilitates signaling the temporal cues to enhance motor preparatory processes for sound synchronization (Fujioka et al., 2012), such that dancers who have training in predictive actions and moving in synchrony with an auditory rhythm (Fujioka et al., 2012) might have facilitation in these processes (Fujioka et al., 2015). These studies underpin isolating song and dance training in research on instrumental musical experience in AV music perception as they might have confounding effects on brain regions, such as the motor cortex, auditory cortex, and superior temporal sulcus (Arnal et al., 2009; Arnal and Giraud, 2012), as well as the role of beta oscillation in sensory-motor tasks (Fujioka et al., 2009).

Previous research has shown that playing a musical instrument involves continual cooperative processing between the visual, auditory, and sensory-motor networks, both in terms of motor timing and motor planning (Zatorre et al., 2007). While playing an instrument, musicians usually integrate sensory information from sight-reading (Sluming et al., 2007) and hand and finger movements, with auditory feedback from the musical sound of the instrument they are playing (Baumann et al., 2007; Jamali et al., 2014), as well as visual feedback from seeing their hand and finger movements while playing the instrument (Richardson et al., 2013). Moreover, the presentation of AV (Gordon et al., 2018b) or auditory music is sufficient to activate motor networks (Gordon et al., 2018a). Therefore, musical experience, which requires such cross-modal coordination, may improve timing and execution over many years of musical training (Zatorre et al., 2007; Jamali et al., 2014).

Previous studies on musical experience (e.g., Kühnis et al., 2014) have suggested that musicians, compared to non-musicians, have increased beta activity. Moreover, research (e.g., Lalo et al., 2007; Patel and Iversen, 2014) further suggests that beta oscillation underpins functional pairing of distant cortical areas, such as the motor, visual (Comstock et al., 2018), and auditory cortex. Beta oscillation supports sensory-motor integration and feedback loops (Arnal, 2012), allowing coordinated task-related modulation of auditory and motor processing (Lalo et al., 2007). For example, beta desynchronization is involved in self-paced motor tasks (Gilbertson et al., 2005; Senkowski et al., 2006). Moreover, beta activity inversely correlated with response time in an AV task (Senkowski et al., 2006), and reflects timing predictions within the visual system (Comstock et al., 2018), suggesting that visual predictory processing may be involved in beta oscillation for AV perception. Findings in the current study highlight that more needs to be understood about the role of beta oscillation and visual perception. Together, these studies suggest that lower beta activity in AV perception compared to the auditory music perception for musicians might be associated with the potential involvement of beta oscillation in auditory-motor tasks, even with no intention for movement (Fujioka et al., 2009; Fujioka and Ross, 2017). This would suggest that for musicians, AV perception is modulated by activating auditory and sensory-motor, possibly also visual, networks over years of instrumental training (Trainor et al., 2009).

## 5. Conclusions

This study supports previous ERP research on AV modulation, suggesting that the predictory visual cues from hand and finger movements starting before the auditory onset of musical sound lead to lower N1 amplitude and latency and lower P2 amplitude independent of musical training. These findings are consistent with previous research with speech and non-speech stimuli suggesting similar AV modulation in early sensory processing. The proposal that AV may be broadly correlated with auditory perception (Baart, 2016), extended to music perception in the current study, is generally supported by the ERP findings. Notably, differences for N1 amplitude between musicians and non-musicians further indicate musical experience to be a specific factor influencing AV perception.

A novel contribution of this research is investigating the predictory effect of visual cues starting before the musical sound onset by ITPC analysis. Coinciding with the amplitude suppression for early ERPs amplitude, ITPC values in corresponding frequency activity were lower in AV music perception compared to the auditory music perception, regardless of musical experience. However, beta activity differed with musical training; while musicians showed lower beta ITPCs in AV, compared to the auditory music perception, non-musicians did not. These findings suggest an association between beta activity in AV music perception with beta oscillation in sensory-motor tasks, as well as visual predictory processing. Moreover, for musicians, visual predictory processes in AV music perception have been influenced by years of multisensory training, which appears to also modulate beta-band activity. This study highlights the possible interconnectivity in AV perception as well as the potential modulation with experience.

## Data Availability Statement

The datasets generated for this study are available on request to the corresponding author.

## Ethics Statement

The studies involving human participants were reviewed and approved by Norwegian Center for Research Data. The patients/participants provided their written informed consent to participate in this study.

## Author Contributions

Both authors contributed extensively to the work presented in this paper. MS and DB jointly conceived of the study and sketched the design. MS carried out the practical implementation of the project, carried out the EEG experiments and data analyses, and drafted the full paper. DB supervised all stages of the project. Both authors discussed the results and implications and contributed to the manuscript.

## Conflict of Interest

The authors declare that the research was conducted in the absence of any commercial or financial relationships that could be construed as a potential conflict of interest.

## References

[B1] Arnal L. H. (2012). Predicting “when” using the motor system's beta-band oscillations. Front. Hum. Neurosci. 6:225. 10.3389/fnhum.2012.00225 22876228 PMC3410664

[B2] Arnal L. H. Giraud A.-L. (2012). Cortical oscillations and sensory predictions. Trends Cogn. Sci. 16, 390–398. 10.1016/j.tics.2012.05.003 22682813

[B3] Arnal L. H. Morillon B. Kell C. A. Giraud A.-L. (2009). Dual neural routing of visual facilitation in speech processing. J. Neurosci. 29, 13445–13453. 10.1523/JNEUROSCI.3194-09.2009 19864557 PMC6665008

[B4] Arnal L. H. Poeppel D. Giraud A.-L. (2015). Temporal coding in the auditory cortex, in Handbook of Clinical Neurology, Vol. 129 (Elsevier), 85–98. 10.1016/B978-0-444-62630-1.00005-6 25726264

[B5] Arnal L. H. Wyart V. Giraud A.-L. (2011). Transitions in neural oscillations reflect prediction errors generated in audiovisual speech. Nat. Neurosci. 14:797. 10.1038/nn.2810 21552273

[B6] Baart M. (2016). Quantifying lip-read-induced suppression and facilitation of the auditory n1 and p2 reveals peak enhancements and delays. Psychophysiology 53, 1295–1306. 10.1111/psyp.12683 27295181

[B7] Baart M. Samuel A. G. (2015). Early processing of auditory lexical predictions revealed by ERPs. Neurosci. Lett. 585, 98–102. 10.1016/j.neulet.2014.11.044 25438158

[B8] Baart M. Stekelenburg J. J. Vroomen J. (2014). Electrophysiological evidence for speech-specific audiovisual integration. Neuropsychologia 53, 115–121. 10.1016/j.neuropsychologia.2013.11.011 24291340

[B9] Baker S. N. (2007). Oscillatory interactions between sensorimotor cortex and the periphery. Curr. Opin. Neurobiol. 17, 649–655. 10.1016/j.conb.2008.01.007 18339546 PMC2428102

[B10] Barnett M. M. Weaver A. J. Cannon A. R. (2017). Patterns procedural learning for pitch matching in adult musicians and non-musicians. J. Acous. Soc. Am. 142, 2613–2613. 10.1121/1.5014569

[B11] Bastiaansen M. C. Böcker K. B. Brunia C. H. De Munck J. C. Spekreijse H. (2001). Event-related desynchronization during anticipatory attention for an upcoming stimulus: a comparative EEG/MEG study. Clin. Neurophysiol. 112, 393–403. 10.1016/S1388-2457(00)00537-X 11165546

[B12] Bastiaansen M. C. Brunia C. H. (2001). Anticipatory attention: an event-related desynchronization approach. Int. J. Psychophysiol. 43, 91–107. 10.1016/S0167-8760(01)00181-7 11742687

[B13] Baumann S. Koeneke S. Schmidt C. F. Meyer M. Lutz K. Jancke L. (2007). A network for audio-motor coordination in skilled pianists and non-musicians. Brain Res. 1161, 65–78. 10.1016/j.brainres.2007.05.045 17603027

[B14] Baumann S. Meyer M. Jäncke L. (2008). Enhancement of auditory-evoked potentials in musicians reflects an influence of expertise but not selective attention. J. Cogn. Neurosci. 20, 2238–2249. 10.1162/jocn.2008.20157 18457513

[B15] Behne K.-E. Wöllner C. (2011). Seeing or hearing the pianists? A synopsis of an early audiovisual perception experiment and a replication. Music. Sci. 15, 324–342. 10.1177/1029864911410955

[B16] Besle J. Fort A. Delpuech C. Giard M.-H. (2004). Bimodal speech: early suppressive visual effects in human auditory cortex. Eur. J. Neurosci. 20, 2225–2234. 10.1111/j.1460-9568.2004.03670.x 15450102 PMC1885424

[B17] Bhattacharya J. Petsche H. (2005). Phase synchrony analysis of EEG during music perception reveals changes in functional connectivity due to musical expertise. Signal Process. 85, 2161–2177. 10.1016/j.sigpro.2005.07.007

[B18] Bianchi F. Hjortkjær J. Santurette S. Zatorre R. J. Siebner H. R. Dau T. (2017). Subcortical and cortical correlates of pitch discrimination: evidence for two levels of neuroplasticity in musicians. Neuroimage 163, 398–412. 10.1016/j.neuroimage.2017.07.057 28774646

[B19] Biau E. Soto-Faraco S. (2015). Synchronization by the hand: the sight of gestures modulates low-frequency activity in brain responses to continuous speech. Front. Hum. Neurosci. 9:527. 10.3389/fnhum.2015.00527 26441618 PMC4585072

[B20] Bidelman G. M. (2017). Amplified induced neural oscillatory activity predicts musicians' benefits in categorical speech perception. Neuroscience 348, 107–113. 10.1016/j.neuroscience.2017.02.015 28214576

[B21] Bidelman G. M. Weiss M. W. Moreno S. Alain C. (2014). Coordinated plasticity in brainstem and auditory cortex contributes to enhanced categorical speech perception in musicians. Eur. J. Neurosci. 40, 2662–2673. 10.1111/ejn.12627 24890664

[B22] Bosnyak D. J. Eaton R. A. Roberts L. E. (2004). Distributed auditory cortical representations are modified when non-musicians are trained at pitch discrimination with 40 Hz amplitude modulated tones. Cereb. Cortex 14, 1088–1099. 10.1093/cercor/bhh068 15115745

[B23] Busch N. A. VanRullen R. (2010). Spontaneous EEG oscillations reveal periodic sampling of visual attention. Proc. Natl. Acad. Sci. U.S.A. 107, 16048–16053. 10.1073/pnas.1004801107 20805482 PMC2941320

[B24] Buzsáki G. Draguhn A. (2004). Neuronal oscillations in cortical networks. Science 304, 1926–1929. 10.1126/science.1099745 15218136

[B25] Canolty R. T. Knight R. T. (2010). The functional role of cross-frequency coupling. Trends Cogn. Sci. 14, 506–515. 10.1016/j.tics.2010.09.001 20932795 PMC3359652

[B26] Cohen M. X. (2014). Analyzing Neural Time Series Data: Theory and Practice. London: MIT press 10.7551/mitpress/9609.001.0001

[B27] Comstock D. C. Hove M. J. Balasubramaniam R. (2018). Sensorimotor synchronization with auditory and visual modalities: behavioral and neural differences. Front. Comput. Neurosci. 12:53. 10.3389/fncom.2018.00053 30072885 PMC6058047

[B28] Cravo A. M. Rohenkohl G. Wyart V. Nobre A. C. (2011). Endogenous modulation of low frequency oscillations by temporal expectations. J. Neurophysiol. 106, 2964–2972. 10.1152/jn.00157.2011 21900508 PMC3234094

[B29] Delorme A. Makeig S. (2004). EEGLAB: an open source toolbox for analysis of single-trial EEG dynamics including independent component analysis. J. Neurosci. methods 134, 9–21. 10.1016/j.jneumeth.2003.10.009 15102499

[B30] Doelling K. B. Arnal L. H. Ghitza O. Poeppel D. (2014). Acoustic landmarks drive delta-theta oscillations to enable speech comprehension by facilitating perceptual parsing. Neuroimage 85, 761–768. 10.1016/j.neuroimage.2013.06.035 23791839 PMC3839250

[B31] Doelling K. B. Assaneo M. F. Bevilacqua D. Pesaran B. Poeppel D. (2019). An oscillator model better predicts cortical entrainment to music. Proc. Natl. Acad. Sci. U.S.A. 116, 10113–10121. 10.1073/pnas.1816414116 31019082 PMC6525506

[B32] Doelling K. B. Poeppel D. (2015). Cortical entrainment to music and its modulation by expertise. Proc. Natl. Acad. Sci. U.S.A. 112, E6233–E6242. 10.1073/pnas.1508431112 26504238 PMC4653203

[B33] Edwards E. Soltani M. Kim W. Dalal S. S. Nagarajan S. S. Berger M. S.. (2009). Comparison of time-frequency responses and the event-related potential to auditory speech stimuli in human cortex. J. Neurophysiol. 102, 377–386. 10.1152/jn.90954.2008 19439673 PMC2712274

[B34] Eggermont J. J. (2007). Correlated neural activity as the driving force for functional changes in auditory cortex. Hear. Res. 229, 69–80. 10.1016/j.heares.2007.01.008 17296278

[B35] Engel A. K. Fries P. (2010). Beta-band oscillations' signalling the status quo? Curr. Opin. Neurobiol. 20, 156–165. 10.1016/j.conb.2010.02.015 20359884

[B36] Foxe J. J. Snyder A. C. (2011). The role of alpha-band brain oscillations as a sensory suppression mechanism during selective attention. Front. Psychol. 2:154. 10.3389/fpsyg.2011.00154 21779269 PMC3132683

[B37] Fuentemilla L. Marco-Pallarés J. Grau C. (2006). Modulation of spectral power and of phase resetting of EEG contributes differentially to the generation of auditory event-related potentials. Neuroimage 30, 909–916. 10.1016/j.neuroimage.2005.10.036 16376575

[B38] Fujioka T. Ross B. (2017). Beta-band oscillations during passive listening to metronome sounds reflect improved timing representation after short-term musical training in healthy older adults. Eur. J. Neurosci. 46, 2339–2354. 10.1111/ejn.13693 28887898

[B39] Fujioka T. Ross B. Trainor L. J. (2015). Beta-band oscillations represent auditory beat and its metrical hierarchy in perception and imagery. J. Neurosci. 35, 15187–15198. 10.1523/JNEUROSCI.2397-15.2015 26558788 PMC6605356

[B40] Fujioka T. Trainor L. Large E. Ross B. (2009). Beta and gamma rhythms in human auditory cortex during musical beat processing. Ann. N. Y. Acad. Sci. 1169, 89–92. 10.1111/j.1749-6632.2009.04779.x 19673759

[B41] Fujioka T. Trainor L. J. Large E. W. Ross B. (2012). Internalized timing of isochronous sounds is represented in neuromagnetic beta oscillations. J. Neurosci. 32, 1791–1802. 10.1523/JNEUROSCI.4107-11.2012 22302818 PMC6703342

[B42] Gilbertson T. Lalo E. Doyle L. Di Lazzaro V. Cioni B. Brown P. (2005). Existing motor state is favored at the expense of new movement during 13-35 Hz oscillatory synchrony in the human corticospinal system. J. Neurosci. 25, 7771–7779. 10.1523/JNEUROSCI.1762-05.2005 16120778 PMC6725263

[B43] Giraud A.-L. Poeppel D. (2012). Cortical oscillations and speech processing: emerging computational principles and operations. Nat. Neurosci. 15:511. 10.1038/nn.3063 22426255 PMC4461038

[B44] Gisladottir R. S. Bögels S. Levinson S. C. (2018). Oscillatory brain responses reflect anticipation during comprehension of speech acts in spoken dialog. Front. Hum. Neurosci. 12:34. 10.3389/fnhum.2018.00034 29467635 PMC5808328

[B45] Gordon C. L. Cobb P. R. Balasubramaniam R. (2018a). Recruitment of the motor system during music listening: an ale meta-analysis of fMRI data. PLoS ONE 13:e0207213. 10.1371/journal.pone.0207213 30452442 PMC6242316

[B46] Gordon C. L. Iacoboni M. Balasubramaniam R. (2018b). Multimodal music perception engages motor prediction: a TMS study. Front. Neurosci. 12:736. 10.3389/fnins.2018.00736 30405332 PMC6201045

[B47] Gruber W. R. Klimesch W. Sauseng P. Doppelmayr M. (2004). Alpha phase synchronization predicts p1 and n1 latency and amplitude size. Cereb. Cortex 15, 371–377. 10.1093/cercor/bhh139 15749980

[B48] Halwani G. F. Loui P. Rueber T. Schlaug G. (2011). Effects of practice and experience on the arcuate fasciculus: comparing singers, instrumentalists, and non-musicians. Front. Psychol. 2:156. 10.3389/fpsyg.2011.00156 21779271 PMC3133864

[B49] Hänggi J. Koeneke S. Bezzola L. Jäncke L. (2010). Structural neuroplasticity in the sensorimotor network of professional female ballet dancers. Hum. Brain Mapp. 31, 1196–1206. 10.1002/hbm.20928 20024944 PMC6870845

[B50] Haslinger B. Erhard P. Altenmüller E. Schroeder U. Boecker H. Ceballos-Baumann A. O. (2005). Transmodal sensorimotor networks during action observation in professional pianists. J. Cogn. Neurosci. 17, 282–293. 10.1162/0898929053124893 15811240

[B51] Heggli O. A. Konvalinka I. Kringelbach M. L. Vuust P. (2019). Musical interaction is influenced by underlying predictive models and musical expertise. Sci. Rep. 9, 1–13. 10.1038/s41598-019-47471-3 31363106 PMC6667437

[B52] Howard M. F. Poeppel D. (2012). The neuromagnetic response to spoken sentences: co-modulation of theta band amplitude and phase. Neuroimage 60, 2118–2127. 10.1016/j.neuroimage.2012.02.028 22374481 PMC3593735

[B53] Hsu Y.-F. Hämäläinen J. A. Waszak F. (2016). The auditory n1 suppression rebounds as prediction persists over time. Neuropsychologia 84, 198–204. 10.1016/j.neuropsychologia.2016.02.019 26921479

[B54] Huhn Z. Szirtes G. Lőrincz A. Csépe V. (2009). Perception based method for the investigation of audiovisual integration of speech. Neurosci. Lett. 465, 204–209. 10.1016/j.neulet.2009.08.077 19733215

[B55] Jamali S. Fujioka T. Ross B. (2014). Neuromagnetic beta and gamma oscillations in the somatosensory cortex after music training in healthy older adults and a chronic stroke patient. Clin. Neurophysiol. 125, 1213–1222. 10.1016/j.clinph.2013.10.045 24290848

[B56] Jasper H. H. (1958). The ten-twenty electrode system of the international federation. Electroencephalogr. Clin. Neurophysiol. 10, 370–375. 10590970

[B57] Keller A. S. Payne L. Sekuler R. (2017). Characterizing the roles of alpha and theta oscillations in multisensory attention. Neuropsychologia 99, 48–63. 10.1016/j.neuropsychologia.2017.02.021 28259771 PMC5410970

[B58] Kishon-Rabin L. Amir O. Vexler Y. Zaltz Y. (2001). Pitch discrimination: are professional musicians better than non-musicians? J. Basic Clin. Physiol. Pharmacol. 12, 125–144. 10.1515/JBCPP.2001.12.2.125 11605682

[B59] Klucharev V. Möttönen R. Sams M. (2003). Electrophysiological indicators of phonetic and non-phonetic multisensory interactions during audiovisual speech perception. Cogn. Brain Res. 18, 65–75. 10.1016/j.cogbrainres.2003.09.004 14659498

[B60] Koelsch S. Vuust P. Friston K. (2019). Predictive processes and the peculiar case of music. Trends Cogn. Sci. 23, 63–77. 10.1016/j.tics.2018.10.006 30471869

[B61] Koerner T. K. Zhang Y. (2015). Effects of background noise on inter-trial phase coherence and auditory n1-p2 responses to speech stimuli. Hear. Res. 328, 113–119. 10.1016/j.heares.2015.08.002 26276419

[B62] Kühnis J. Elmer S. Jäncke L. (2014). Auditory evoked responses in musicians during passive vowel listening are modulated by functional connectivity between bilateral auditory-related brain regions. J. Cogn. Neurosci. 26, 2750–2761. 10.1162/jocn_a_00674 24893742

[B63] Kühnis J. Elmer S. Meyer M. Jäncke L. (2013). The encoding of vowels and temporal speech cues in the auditory cortex of professional musicians: an EEG study. Neuropsychologia 51, 1608–1618. 10.1016/j.neuropsychologia.2013.04.007 23664833

[B64] Kuriki S. Kanda S. Hirata Y. (2006). Effects of musical experience on different components of meg responses elicited by sequential piano-tones and chords. J. Neurosci. 26, 4046–4053. 10.1523/JNEUROSCI.3907-05.2006 16611821 PMC6673882

[B65] Lakatos P. Chen C.-M. O'Connell M. N. Mills A. Schroeder C. E. (2007). Neuronal oscillations and multisensory interaction in primary auditory cortex. Neuron 53, 279–292. 10.1016/j.neuron.2006.12.011 17224408 PMC3717319

[B66] Lalo E. Gilbertson T. Doyle L. Di Lazzaro V. Cioni B. Brown P. (2007). Phasic increases in cortical beta activity are associated with alterations in sensory processing in the human. Exp. Brain Res. 177, 137–145. 10.1007/s00221-006-0655-8 16972074

[B67] Lange J. Christian N. Schnitzler A. (2013). Audio-visual congruency alters power and coherence of oscillatory activity within and between cortical areas. Neuroimage 79, 111–120. 10.1016/j.neuroimage.2013.04.064 23644355

[B68] Lange K. (2013). The ups and downs of temporal orienting: a review of auditory temporal orienting studies and a model associating the heterogeneous findings on the auditory n1 with opposite effects of attention and prediction. Front. Hum. Neurosci. 7:263. 10.3389/fnhum.2013.00263 23781186 PMC3678089

[B69] Large E. Snyder J. (2009). Pulse and meter as neural resonance. Ann. N. Y. Acad. Sci. 1169, 46–57. 10.1111/j.1749-6632.2009.04550.x 19673754

[B70] Lee H. Noppeney U. (2011). Long-term music training tunes how the brain temporally binds signals from multiple senses. Proc. Natl. Acad. Sci. U.S.A. 108:E1441–E1450. 10.1073/pnas.1115267108 22114191 PMC3251069

[B71] Liem F. Zaehle T. Burkhard A. Jäncke L. Meyer M. (2012). Cortical thickness of supratemporal plane predicts auditory n1 amplitude. Neuroreport 23, 1026–1030. 10.1097/WNR.0b013e32835abc5c 23076120

[B72] Luck S. J. (2014). An introduction to the event-related potential technique. London: MIT press.

[B73] Luo H. Poeppel D. (2012). Cortical oscillations in auditory perception and speech: evidence for two temporal windows in human auditory cortex. Front. Psychol. 3:170. 10.3389/fpsyg.2012.00170 22666214 PMC3364513

[B74] Lütkenhöner B. Seither-Preisler A. Seither S. (2006). Piano tones evoke stronger magnetic fields than pure tones or noise, both in musicians and non-musicians. Neuroimage 30, 927–937. 10.1016/j.neuroimage.2005.10.034 16337814

[B75] Maes P.-J. Leman M. Palmer C. Wanderley M. (2014). Action-based effects on music perception. Front. Psychol. 4:1008. 10.3389/fpsyg.2013.01008 24454299 PMC3879531

[B76] Maslennikova A. Varlamov A. Strelets V. (2015). Characteristics of evoked changes in EEG spectral power and evoked potentials on perception of musical harmonies in musicians and nonmusicians. Neurosci. Behav. Physiol. 45, 78–83. 10.1007/s11055-014-0042-z

[B77] Mikutta C. Maissen G. Altorfer A. Strik W. König T. (2014). Professional musicians listen differently to music. Neuroscience 268, 102–111. 10.1016/j.neuroscience.2014.03.007 24637097

[B78] Molnar-Szakacs I. Overy K. (2006). Music and mirror neurons: from motion to ‘e” motion. Soc. Cogn. Affect. Neurosci. 1, 235–241. 10.1093/scan/nsl029 18985111 PMC2555420

[B79] Musacchia G. Strait D. Kraus N. (2008). Relationships between behavior, brainstem and cortical encoding of seen and heard speech in musicians and non-musicians. Hear. Res. 241, 34–42. 10.1016/j.heares.2008.04.013 18562137 PMC2701624

[B80] Näätänen R. Kujala T. Winkler I. (2011). Auditory processing that leads to conscious perception: a unique window to central auditory processing opened by the mismatch negativity and related responses. Psychophysiology 48, 4–22. 10.1111/j.1469-8986.2010.01114.x 20880261

[B81] Näätänen R. Picton T. (1987). The n1 wave of the human electric and magnetic response to sound: a review and an analysis of the component structure. Psychophysiology 24, 375–425. 10.1111/j.1469-8986.1987.tb00311.x 3615753

[B82] Näätänen R. Winkler I. (1999). The concept of auditory stimulus representation in cognitive neuroscience. Psychol. Bull. 125:826. 10.1037/0033-2909.125.6.826 10589304

[B83] Oldfield R. C. (1971). The assessment and analysis of handedness: the edinburgh inventory. Neuropsychologia 9, 97–113. 10.1016/0028-3932(71)90067-4 5146491

[B84] Panasiti M. Pavone E. Aglioti S. (2016). Electrocortical signatures of detecting errors in the actions of others: an EEG study in pianists, non-pianist musicians and musically naïve people. Neuroscience 318, 104–113. 10.1016/j.neuroscience.2016.01.023 26777892

[B85] Pantev C. Roberts L. E. Schulz M. Engelien A. Ross B. (2001). Timbre-specific enhancement of auditory cortical representations in musicians. Neuroreport 12, 169–174. 10.1097/00001756-200101220-00041 11201080

[B86] Paraskevopoulos E. Kuchenbuch A. Herholz S. C. Pantev C. (2012). Musical expertise induces audiovisual integration of abstract congruency rules. J. Neurosci. 32, 18196–18203. 10.1523/JNEUROSCI.1947-12.2012 23238733 PMC6621720

[B87] Paris T. Kim J. Davis C. (2013). Visual speech form influences the speed of auditory speech processing. Brain Lang. 126, 350–356. 10.1016/j.bandl.2013.06.008 23942046

[B88] Paris T. Kim J. Davis C. (2016a). The processing of attended and predicted sounds in time. J. Cogn. Neurosci. 28, 158–165. 10.1162/jocn_a_00885 26439266

[B89] Paris T. Kim J. Davis C. (2016b). Using EEG and stimulus context to probe the modelling of auditory-visual speech. Cortex 75, 220–230. 10.1016/j.cortex.2015.03.010 26045213

[B90] Paris T. Kim J. Davis C. (2017). Visual form predictions facilitate auditory processing at the n1. Neuroscience 343, 157–164. 10.1016/j.neuroscience.2016.09.023 27646290

[B91] Patel A. D. (2011). Why would musical training benefit the neural encoding of speech? The opera hypothesis. Front. Psychol. 2:142. 10.3389/fpsyg.2011.00142 21747773 PMC3128244

[B92] Patel A. D. Iversen J. R. (2014). The evolutionary neuroscience of musical beat perception: the action simulation for auditory prediction (ASAP) hypothesis. Front. Syst. Neurosci. 8:57. 10.3389/fnsys.2014.00057 24860439 PMC4026735

[B93] Pelli D. G. Vision S. (1997). The videotoolbox software for visual psychophysics: transforming numbers into movies. Spatial Vis. 10, 437–442. 10.1163/156856897X00366 9176953

[B94] Petrini K. Dahl S. Rocchesso D. Waadeland C. H. Avanzini F. Puce A.. (2009a). Multisensory integration of drumming actions: musical expertise affects perceived audiovisual asynchrony. Exp. Brain Res. 198:339. 10.1007/s00221-009-1817-2 19404620

[B95] Petrini K. Pollick F. E. Dahl S. McAleer P. McKay L. Rocchesso D.. (2011). Action expertise reduces brain activity for audiovisual matching actions: an fMRI study with expert drummers. Neuroimage 56, 1480–1492. 10.1016/j.neuroimage.2011.03.009 21397699

[B96] Petrini K. Russell M. Pollick F. (2009b). When knowing can replace seeing in audiovisual integration of actions. Cognition 110, 432–439. 10.1016/j.cognition.2008.11.015 19121519

[B97] Pilling M. (2009). Auditory event-related potentials (ERPs) in audiovisual speech perception. J. Speech Lang. Hear. Res. 52, 1073–1081. 10.1044/1092-4388(2009/07-0276) 19641083

[B98] Platz F. Kopiez R. (2012). When the eye listens: a meta-analysis of how audio-visual presentation enhances the appreciation of music performance. Music Percept. Interdiscipl. J. 30, 71–83. 10.1525/mp.2012.30.1.71

[B99] Poikonen H. Toiviainen P. Tervaniemi M. (2018). Dance on cortex: enhanced theta synchrony in experts when watching a dance piece. Eur. J. Neurosci. 47, 433–445. 10.1111/ejn.13838 29359365

[B100] Pratt H. (2011). Sensory ERP components, in The Oxford Handbook of Event-Related Potential Components, eds Luck S. J. Kappenman E. S. (New York, NY: Oxford University Press Inc.), 89–114. 10.1093/oxfordhb/9780195374148.013.0050

[B101] Proverbio A. M. Massetti G. Rizzi E. Zani A. (2016). Skilled musicians are not subject to the Mcgurk effect. Sci. Rep. 6:30423. 10.1038/srep30423 27453363 PMC4958963

[B102] Repp B. H. Knoblich G. (2009). Performed or observed keyboard actions affect pianists' judgements of relative pitch. Q. J. Exp. Psychol. 62, 2156–2170. 10.1080/17470210902745009 19358057

[B103] Richardson B. A. Cluff T. Lyons J. Balasubramaniam R. (2013). An eye-to-hand magnet effect reveals distinct spatial interference in motor planning and execution. Exp. Brain Res. 225, 443–454. 10.1007/s00221-012-3384-1 23417695

[B104] Rigoulot S. Pell M. D. Armony J. L. (2015). Time course of the influence of musical expertise on the processing of vocal and musical sounds. Neuroscience. 290, 175–184. 10.1016/j.neuroscience.2015.01.033 25637804

[B105] Sanju H. K. Kumar P. (2016). Enhanced auditory evoked potentials in musicians: A review of recent findings. J. Otol. 11, 63–72. 10.1016/j.joto.2016.04.002 29937812 PMC6002589

[B106] Schön D. Magne C. Besson M. (2004). The music of speech: music training facilitates pitch processing in both music and language. Psychophysiol. 41, 341–349. 10.1111/1469-8986.00172.x 15102118

[B107] Schroeder C. E. Lakatos P. (2009). Low-frequency neuronal oscillations as instruments of sensory selection. Trends Neurosci. 32, 9–18. 10.1016/j.tins.2008.09.012 19012975 PMC2990947

[B108] Schwartz J.-L. Berthommier F. Savariaux C. (2004). Seeing to hear better: evidence for early audio-visual interactions in speech identification. Cognition 93, B69–B78. 10.1016/j.cognition.2004.01.006 15147940

[B109] Sebanz N. Knoblich G. (2009). Prediction in joint action: what, when, and where. Top. Cogn. Sci. 1, 353–367. 10.1111/j.1756-8765.2009.01024.x 25164938

[B110] Senkowski D. Molholm S. Gomez-Ramirez M. Foxe J. J. (2005). Oscillatory beta activity predicts response speed during a multisensory audiovisual reaction time task: a high-density electrical mapping study. Cereb. Cortex 16, 1556–1565. 10.1093/cercor/bhj091 16357336

[B111] Senkowski D. Molholm S. Gomez-Ramirez M. Foxe J. J. (2006). Oscillatory beta activity predicts response speed during a multisensory audiovisual reaction time task: a high-density electrical mapping study. Cereb. Cortex 16, 1556–1565. 16357336 10.1093/cercor/bhj091

[B112] Shahin A. Bosnyak D. J. Trainor L. J. Roberts L. E. (2003). Enhancement of neuroplastic p2 and n1c auditory evoked potentials in musicians. J. Neurosci. 23, 5545–5552. 10.1523/JNEUROSCI.23-13-05545.2003 12843255 PMC6741225

[B113] Shahin A. Roberts L. E. Pantev C. Trainor L. J. Ross B. (2005). Modulation of p2 auditory-evoked responses by the spectral complexity of musical sounds. Neuroreport 16, 1781–1785. 10.1097/01.wnr.0000185017.29316.63 16237326

[B114] Sluming V. Brooks J. Howard M. Downes J. J. Roberts N. (2007). Broca's area supports enhanced visuospatial cognition in orchestral musicians. J. Neurosci. 27, 3799–3806. 10.1523/JNEUROSCI.0147-07.2007 17409244 PMC6672394

[B115] Sorati M. Behne D. M. (2019). Musical expertise affects audiovisual speech perception: Findings from event-related potentials and inter-trial phase coherence. Front. Psychol. 10:2562. 10.3389/fpsyg.2019.02562 31803107 PMC6874039

[B116] Stefanics G. Hangya B. Hernádi I. Winkler I. Lakatos P. Ulbert I. (2010). Phase entrainment of human delta oscillations can mediate the effects of expectation on reaction speed. J. Neurosci. 30, 13578–13585. 10.1523/JNEUROSCI.0703-10.2010 20943899 PMC4427664

[B117] Stekelenburg J. Vroomen J. (2012). Electrophysiological correlates of predictive coding of auditory location in the perception of natural audiovisual events. Front. Integr. Neurosci. 6:26. 10.3389/fnint.2012.00026 22666195 PMC3364694

[B118] Stekelenburg J. J. Vroomen J. (2007). Neural correlates of multisensory integration of ecologically valid audiovisual events. J. Cogn. Neurosci. 19, 1964–1973. 10.1162/jocn.2007.19.12.1964 17892381

[B119] Strait D. L. Kraus N. (2014). Biological impact of auditory expertise across the life span: musicians as a model of auditory learning. Hear. Res. 308, 109–121. 10.1016/j.heares.2013.08.004 23988583 PMC3947192

[B120] Strauß A. Wöstmann M. Obleser J. (2014). Cortical alpha oscillations as a tool for auditory selective inhibition. Front. Hum. Neurosci. 8:350. 10.3389/fnhum.2014.00350 24904385 PMC4035601

[B121] Tan A. Hu L. Tu Y. Chen R. Hung Y. S. Zhang Z. (2016). N1 magnitude of auditory evoked potentials and spontaneous functional connectivity between bilateral heschl's gyrus are coupled at interindividual level. Brain Connect. 6, 496–504. 10.1089/brain.2016.0418 27105665

[B122] Thompson W. F. Graham P. Russo F. A. (2005). Seeing music performance: visual influences on perception and experience. Semiotica 2005, 203–227. 10.1515/semi.2005.2005.156.203

[B123] Todorovic A. Schoffelen J.-M. van Ede F. Maris E. de Lange F. P. (2015). Temporal expectation and attention jointly modulate auditory oscillatory activity in the beta band. PLoS ONE 10:e0120288. 10.1371/journal.pone.0120288 25799572 PMC4370604

[B124] Trainor L. J. Shahin A. J. Robertsa L. E. (2009). Understanding the benefits of musical training. Neurosci. Music III Disord. Plastic. 1169:133. 10.1111/j.1749-6632.2009.04589.x 19673769

[B125] Tremblay K. L. Billings C. J. Friesen L. M. Souza P. E. (2006). Neural representation of amplified speech sounds. Ear Hear. 27, 93–103. 10.1097/01.aud.0000202288.21315.bd 16518138

[B126] van Diepen R. M. Mazaheri A. (2018). The caveats of observing inter-trial phase-coherence in cognitive neuroscience. Sci. Rep. 8:2990. 10.1038/s41598-018-20423-z 29445210 PMC5813180

[B127] van Ede F. Szebényi S. Maris E. (2014). Attentional modulations of somatosensory alpha, beta and gamma oscillations dissociate between anticipation and stimulus processing. Neuroimage 97, 134–141. 10.1016/j.neuroimage.2014.04.047 24769186

[B128] van Wassenhove V. (2013). Speech through ears and eyes: interfacing the senses with the supramodal brain. Front. Psychol. 4:388. 10.3389/fpsyg.2013.00388 23874309 PMC3709159

[B129] Van Wassenhove V. Grant K. W. Poeppel D. (2005). Visual speech speeds up the neural processing of auditory speech. Proceedings of the National Academy of Sciences 102, 1181–1186. 10.1073/pnas.0408949102 15647358 PMC545853

[B130] Virtala P. Huotilainen M. Partanen E. Tervaniemi M. (2014). Musicianship facilitates the processing of western music chords–an erp and behavioral study. Neuropsychologia 61, 247–258. 10.1016/j.neuropsychologia.2014.06.028 24992584

[B131] Vroomen J. Stekelenburg J. J. (2010). Visual anticipatory information modulates multisensory interactions of artificial audiovisual stimuli. J. Cogn. Neurosci. 22, 1583–1596. 10.1162/jocn.2009.21308 19583474

[B132] Wild C. J. Yusuf A. Wilson D. E. Peelle J. E. Davis M. H. Johnsrude I. S. (2012). Effortful listening: the processing of degraded speech depends critically on attention. J. Neurosci. 32, 14010–14021. 10.1523/JNEUROSCI.1528-12.2012 23035108 PMC6704770

[B133] Zatorre R. J. Chen J. L. Penhune V. B. (2007). When the brain plays music: auditory-motor interactions in music perception and production. Nat. Rev. Neurosci. 8:547. 10.1038/nrn2152 17585307

